# Fleaborne Typhus–Associated Deaths — Los Angeles County, California, 2022

**DOI:** 10.15585/mmwr.mm7231a1

**Published:** 2023-08-04

**Authors:** Jemma Alarcón, Armine Sanosyan, Zuelma A. Contreras, Van P. Ngo, Ann Carpenter, Jill K. Hacker, William S. Probert, Dawn Terashita, Sharon Balter, Umme-Aiman Halai

**Affiliations:** ^1^Epidemic Intelligence Service, CDC; ^2^Los Angeles County Department of Public Health, Los Angeles, California; ^3^California Department of Public Health.

**Summary**

**What is already known about this topic?**

Fleaborne typhus, a vectorborne zoonosis caused by *Rickettsia* *typhi*, is a moderately severe but rarely fatal illness.

**What is added by this report?**

Fleaborne typhus cases in Los Angeles County (LAC), California increased from 31 in 2010 to 171 in 2022. In 2022, three associated deaths occurred among LAC adults with underlying medical conditions; severe manifestations included hemophagocytic lymphohistiocytosis, myocarditis, and septic shock.

**What are the implications for public health practice?**

Health care providers should suspect fleaborne typhus in patients with compatible symptoms who live in or travel to areas with endemic disease or are exposed to reservoir animals; prompt initiation of doxycycline therapy is critical. Monitoring rodent, opossum, free-roaming cat, and dog flea infestations and the numbers of infected fleas is needed to understand disease ecology and more efficiently direct interventions to prevent disease in humans.

## Abstract

Fleaborne typhus (also known as murine typhus), a widely distributed vectorborne zoonosis caused by *Rickettsia typhi*, is a moderately severe, but infrequently fatal illness; among patients who receive doxycycline, the case-fatality rate is <1%. Fleaborne typhus is a mandated reportable condition in California. Reported fleaborne typhus cases in Los Angeles County have been increasing since 2010, with the highest number (171) reported during 2022. During June–October 2022, Los Angeles County Department of Public Health learned of three fleaborne typhus–associated deaths. This report describes the clinical presentation, illness course, and methods used to diagnose fleaborne typhus in these three cases. Severe fleaborne typhus manifestations among these cases included hemophagocytic lymphohistiocytosis, a rare immune hyperactivation syndrome that can occur in the infection setting; myocarditis; and septic shock with disseminated intravascular coagulation. Increased health care provider and public health awareness of the prevalence and severity of fleaborne typhus and of the importance of early doxycycline therapy is essential for prevention and treatment efforts.

## Introduction

Fleaborne typhus is transmitted from infected fleas by inoculation of flea feces into the flea bite site, a skin abrasion, or mucous membranes (*1*). The Oriental rat flea (*X*e*nopsylla cheopis*), a parasite of rats, is the historical vector (*2*). The cat flea (*Ctenocephalides felis*), whose principal host is the domestic cat (but which is also found on opossums, dogs, and rats) is the predominant vector in suburban areas of the United States* (*3*). Signs and symptoms of fleaborne typhus include fever, headache, a palm- and sole-sparing rash, hepatitis, and thrombocytopenia (*4*). Approximately one third of infected patients require intensive care for associated aseptic meningitis, seizures, adult respiratory distress syndrome, or septic shock (*4*); however, among patients who receive doxycycline therapy, the case-fatality rate is <1% (*5*). Most current cases in the United States are identified in California, Hawaii, and Texas (*4*). During 1985–2015, among 3,048 fleaborne typhus cases in Texas, 11 (0.4%) were fatal (*6*). The disease is endemic in Los Angeles County (LAC), and reporting is mandated in California (Figure). Before 2022, the most recent fleaborne typhus–associated death in LAC was reported in 1993.

**FIGURE:**
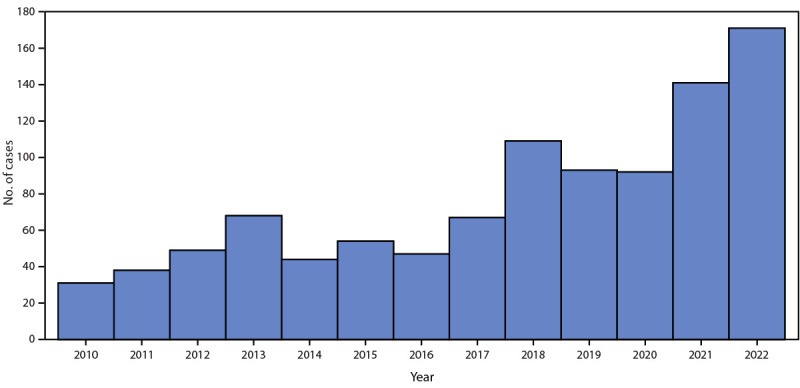
Fleaborne typhus cases, by year — Los Angeles County, California* 2010–2022 * Excluding the cities of Pasadena and Long Beach; data include confirmed, probable, and suspected cases.

## Methods

As part of fleaborne typhus surveillance in LAC, retrospective medical record review and case or next-of-kin interviews are conducted for all reported cases with presumptive or confirmatory laboratory evidence of infection. A presumptive fleaborne typhus case includes detection of *R. typhi* immunoglobulin (Ig) G antibodies at titers ≥1:128 or IgM titers ≥1:256 by indirect immunofluorescence antibody assay obtained from specimens collected within 60 days of illness onset.^†^ Additional testing was performed at the California Department of Public Health Viral and Rickettsial Disease Laboratory for severe or fatal cases, and those in which clinical criteria were met but antibody titers were below case definition thresholds. Two types of real-time polymerase chain reaction (PCR) assays were used, one that identified a 119-base-pair (bp) repeat region within the gene for surface cell antigen 2 (an autotransporter protein), or at the Rickettsial Zoonoses Branch at CDC, an assay that amplified 146-bp or 197-bp fragments of intergenic regions of *R. typhi*.

For the reported deaths in 2022, autopsy findings were reviewed to confirm the cause of death. Testing for *R. typhi* antigens using an immunohistochemical stain was performed on tissues of one patient with fatal fleaborne typhus obtained at autopsy (*7*). This activity was reviewed by CDC and conducted consistent with applicable federal law and CDC policy.^§^

## Results

### Case Series

**Patient A.** In June 2022, a man identifying as Hispanic^¶^ aged 68 years was evaluated in an emergency department (ED) for a 3-day history of fever and progressive lower extremity weakness (Table). Medical history included diffuse lymphadenopathy, obesity, hypertension, diabetes mellitus type 2, and peripheral vascular disease complicated by a chronic left foot ulcer. He had anemia and elevated liver enzymes and was admitted to the hospital with a diagnosis of sepsis and treated with broad-spectrum antibiotics. His mental status deteriorated, and he became difficult to rouse. On hospital day 8, he experienced hypotension and atrial fibrillation with rapid ventricular response and was transferred to the intensive care unit. The next day, he experienced hypoxemic respiratory failure and was placed on mechanical ventilation; the day after, he required vasopressor support and was given stress-dose steroids. On hospital day 9, a bone marrow biopsy was notable for scattered hemophagocytosis (histiocytic phagocytosis of red blood cells, white blood cells, platelets, and their precursors), and on hospital day 16, he received a diagnosis of hemophagocytic lymphohistiocytosis (HLH), a rare immune system disease, for which he received chemotherapy and infection prophylaxis as indicated by HLH-2004 protocol (*8*). He received doxycycline on hospital day 18, after receiving a positive Karius test** result for *R.* *typhi*. On hospital day 24, he no longer required mechanical ventilation, was extubated, and remained minimally responsive. On hospital day 29, he experienced multiorgan failure and transitioned to comfort care; he died on hospital day 30. Death was attributed to fleaborne typhus–induced HLH and septic shock. Potential exposure to rodents and fleas included proximity of the patient’s home to a highway and litter.

**TABLE:** Demographic, epidemiologic, and clinical characteristics of persons who died from fleaborne typhus–related illness — Los Angeles County, California, June–October 2022

Characteristic	Patient		
	A	B	C
**Age, yrs (sex)**	68 (male)	49 (female)	71 (male)
Ethnicity*	Hispanic	Hispanic	Hispanic
Signs and symptoms	Fever for 3 days and progressive lower extremity weakness	Headache, fever, chills, night sweats, and back pain for 7 days	Fever, disorientation, hypotension, AF with rapid ventricular response, and petechial rash (on legs and torso)
Potential exposure	Proximity of the patient’s home to a highway and litter	Stray kittens in patient’s backyard	Lived in an encampment inhabited by persons experiencing homelessness
Underlying medical conditions	Diffuse lymphadenopathy, obesity, hypertension, DM type 2, PVD, and chronic left foot ulcer	Obesity, hypertension, hyperlipidemia, and DM type 2	Alcohol and methamphetamine use
**Abnormal laboratory values (referent range)**			
White blood cell count (4.5–10.0/*μ*l)	—^†^	—^†^	3.4
Immature neutrophils (<10%)	—^†^	—^†^	15
Platelet count (160–360/*μ*l)	—^†^	130	31
Hemoglobin (13.5–16.5/*μ*l)	10.4	—^†^	10.1
Sodium (135–145 mmol/L)	126	—^†^	—^†^
Potassium (3.5–5.1 mmol/L)	—^†^	2.8	—^†^
Magnesium (1.6–2.6 per md/dL)	—^†^	1.5	—^†^
Total bilirubin (<1 mg/dL)	—^†^	—^†^	1
Alanine aminotransferase (10–50 U/L)	143	114	73
Aspartate aminotransferase (10–50 U/L)	102	141	224
Venous lactate (0.5–1.6 mmol/L)	2.6	—^†^	4.8
C-reactive protein (<0.3 mg/L)	—^†^	269.2	—^†^
**Treatments received**	Cefepime, vancomycin, piperacillin-tazobactam, etoposide, dexamethasone, fluconazole, and trimethoprim-sulfamethoxazole	Ceftriaxone, vancomycin, and meropenem	Ceftriaxone, vancomycin, acyclovir, and penicillin
**Doxycycline therapy started, hospital day**	18	2	2
**Major clinical events**	Mental status deterioration, hypotension, AF with rapid ventricular response, hypoxic respiratory failure, HLH, and severe septic shock	SVT, two episodes of cardiac arrest, and multiorgan failure	Hypoxemic respiratory failure, multiorgan failure, and DIC
**Microbiology results**	Epstein-Barr virus infection diagnosed by PCR (hospital days 9 and 16), HSV 1 diagnosed by bronchoscopy specimen, (hospital day 16), multidrug resistant *Escherichia coli* detected in blood cultures, and CMV diagnosed by PCR (hospital day 29)	Parvovirus B19 DNA was detected in blood and heart tissue by PCR	—^†^
***Rickettsia typhi* molecular testing results**			
Titer collection timing, hospital day	19	2	2
Titer result timing, hospital day	28	Patient deceased	Patient deceased
**Titer result**			
IgM titer	>1:256	1:128	1:128
IgG titer	>1:256	1:64	>1:256
**VRDL result**			
IgG titer	Not submitted	>1:1,024	Not submitted
PCR result	Not submitted	Positive	Positive
**Karius test^§^**			
Timing, hospital day	18	Not submitted	Not submitted
Result	Positive for *R. typhi*	—^†^	—^†^
**Days to death after hospitalization**	30	3	5
**Cause of death^¶^**	Fleaborne typhus–induced HLH and septic shock	Myocarditis	Septic shock associated with shock liver, hyperkalemia, and lactic acidosis

**Abbreviations:** AF = atrial fibrillation; CMV = cytomegalovirus; DIC = disseminated intravascular coagulation; DM = diabetes mellitus; HLH = hemophagocytic lymphohistiocytosis; HSV = herpes simplex virus; Ig = immunoglobulin; PCR = real-time polymerase chain reaction; PVD = peripheral vascular disease; SVT = supraventricular tachycardia; VRDL = Viral and Rickettsial Disease Laboratory.

* Persons of Hispanic or Latino (Hispanic) origin might be of any race but are categorized as Hispanic.

^†^ Result within normal limits.

^§^ A noninvasive, rapid cell-free DNA-based diagnostic test capable of identifying bacteria, mycobacteria, DNA viruses, fungi, and protozoa in blood. Host and microbial cell-free DNA isolated from the patient’s blood specimen is sequenced then analyzed using bioinformatics of DNA-based pathogen genomes. The test is useful for identifying rare pathogens. https://kariusdx.com/

^¶^ As recorded on the death certificate.

**Patient B.** In August 2022, a woman identifying as Hispanic aged 49 years was evaluated at an urgent care facility for a 2-day history of headache and fever. Medical history included obesity, hypertension, hyperlipidemia, and diabetes mellitus type 2. During that visit she received a negative SARS-CoV-2 test result and was given a prescription for antihistamines and nasal steroids to treat presumed allergic rhinitis. Five days later, she visited an ED with fever, chills, night sweats, headache, and back pain. She received intravenous fluids and was discharged after symptomatic improvement. She returned to the ED the next day where she was found to be thrombocytopenic, hypokalemic, and had elevated liver enzymes; she was admitted to the hospital with a diagnosis of sepsis; treatment with broad-spectrum antibiotics was initiated. On hospital day 2, she experienced supraventricular tachycardia and two episodes of cardiac arrest with successful resuscitation. Cardiac catheterization found stress cardiomyopathy and no coronary artery disease. In light of the patient’s headache, fever and elevated transaminases, an infectious diseases physician recommended treatment with doxycycline, which was started on hospital day 2, for possible fleaborne typhus. The patient subsequently experienced multiorgan failure and died on hospital day 3. Autopsy confirmed myocarditis as a proximate cause of death. Immunohistochemistry evaluation for typhus group *Rickettsia* demonstrated rare, multifocal staining of rickettsial antigens in endothelial cells in small blood vessels of the heart and less frequently in endothelial cells lining the sinusoidal spaces of the liver (Supplementary Figure, https://stacks.cdc.gov/view/cdc/131262). Potential flea exposure included stray kittens living in the patient’s backyard.

**Patient C.** In October 2022, a man identifying as Hispanic aged 71 years who was experiencing homelessness and had a history of alcohol use disorder was brought to an ED by ambulance after having been observed lying in the same place on the ground for 24 hours. He was febrile, disoriented, hypotensive, tachypneic, and experiencing atrial fibrillation with rapid ventricular response. He had anemia, thrombocytopenia, and a low white blood cell count with a predominance of immature neutrophils, in addition to lactic acidosis and elevated liver enzymes. He had a petechial rash on his legs and torso. Treatment for suspected meningitis, fleaborne typhus, and neurosyphilis was initiated. On hospital day 2, the patient became hypoxemic, and on hospital day 4, experienced hypoxemic respiratory failure and was placed on mechanical ventilation. He experienced worsening multiorgan failure and disseminated intravascular coagulation and transitioned to comfort care; he died on hospital day 5. Causes of death listed on the death certificate were septic shock associated with shock liver, hyperkalemia, and lactic acidosis. The patient might have also been exposed to fleas and rodents at the encampment where he lived.

## Discussion

The identification of three fatal cases of fleaborne typhus in LAC in 2022 occurred in the context of a marked increase in LAC cases in recent years. Texas is also experiencing a substantial increase in the prevalence and geographic distribution of fleaborne typhus (*4*). Although reports of HLH among patients with *R. typhi* infection are rare (*9*), these three fleaborne typhus-associated deaths highlight the range of potentially severe manifestations of this infection, including HLH, myocarditis, and septic shock with disseminated intravascular coagulation. A recent study noted a case-fatality rate of <1% (*6*); in LAC, the case-fatality rate was noted to be 1.8% in 2022. It is likely that given the overall increase in cases, more persons with severe disease and deaths were identified. In addition, all three patients had comorbidities that might have placed them at increased risk for severe disease. A change in the pathogenicity of *R.* *typhi*, although possible, has not been documented and needs to be monitored.

One possible reason for the observed substantial increase in fleaborne typhus cases in suburban areas is the prevalence of the cat flea (*Ctenocephalides felis*), an abundant nonselective parasite vector that affects free-roaming as well as companion animals (*4*). Another possible reason could be an increase in rodent reservoirs in urban and suburban areas in LAC. The fact that fleaborne typhus is no longer a nationally notifiable disease poses surveillance challenges across the United States.^††^ *R. typhi*–induced myocarditis has been reported in areas with endemic transmission (*10*) and should be considered when evaluating a patient with acute coronary syndrome (a condition resulting from a sudden reduction of blood flow to the heart) and an unexplained febrile illness from such an area.

All three fatal cases described in this report had positive *R. typhi* molecular testing results, which confirmed recent fleaborne typhus infection. Commercial *R. typhi* PCR testing is unavailable, and confirmation of fleaborne typhus relies upon evidence of a fourfold increase in IgG antibody titers from acute to postconvalescent illness phases.

### Limitations

The findings in this report are subject to at least two limitations. First, it is likely that only patients with severe disease are tested for *R. typhi*, and surveillance is currently missing patients with milder disease who might not have access to or seek medical care or receive testing for *R.* *typhi* from their health care provider. Second, patients with *R.* *typhi* infections were not followed after they were discharged from the hospital, leading to the possibility that some deaths due to *R.* *typhi* might have been missed.

### Implications for Public Health Practice

No vaccine to prevent fleaborne typhus currently exists. Use of veterinarian-approved flea control products on pets can reduce the risk for flea exposures to humans. Because *R. typhi* testing during early illness might result in nondetectable or low antibody titers, and waiting for convalescent titers inherently delays confirmation of diagnosis, health care providers should initiate treatment with doxycycline as soon as fleaborne typhus is suspected. In addition, health care providers should consider fleaborne typhus in any patient with fever, headache, and rash, particularly if the patient lives in or recently traveled to an area with endemic disease or had exposure to a reservoir animal (e.g., rodents, opossums, or feral cats).^§§^ Monitoring rodent, opossum, cat, and dog flea infestations and the numbers of infected fleas is important to better understand disease ecology and more effectively direct interventions to prevent human disease.

## Footnotes

* http://www.publichealth.lacounty.gov/acd/vectortyphus.htm (Accessed January 25, 2023).

† Fever as reported by the patient or health care provider and two or more of the following signs or symptoms: headache, myalgia, rash, nausea or vomiting, thrombocytopenia, or elevation of hepatic transaminases. http://publichealth.lacounty.gov/acd/Diseases/EpiForms/TyphusRep.pdf

§ 45 C.F.R. part 46.102(l)(2), 21 C.F.R. part 56; 42 U.S.C. Sect. 241(d); 5 U.S.C. Sect/552a; 44 U.S.C. Sect. 3501 et seq.

¶ Persons of Hispanic or Latino (Hispanic) origin might be of any race but are categorized as Hispanic.

** A noninvasive, rapid cell-free DNA-based diagnostic test capable of identifying bacteria, mycobacteria, DNA viruses, fungi, and protozoa in blood. Host and microbial cell-free DNA isolated from the patient’s blood specimen is sequenced then analyzed using bioinformatics of DNA-based pathogen genomes. The test is useful for identifying rare pathogens. https://kariusdx.com/

†† https://www.cdc.gov/typhus/murine/index.html.

§§
https://wwwnc.cdc.gov/travel/yellowbook/2024/infections-diseases/rickettsial-diseases#:~:text
